# Revisiting the relationship between constructive alignment and learning approaches: A perceived alignment perspective

**DOI:** 10.1371/journal.pone.0253949

**Published:** 2021-08-24

**Authors:** Christian Stamov Roßnagel, Katrin Lo Baido, Noleine Fitzallen

**Affiliations:** 1 Department Psychology & Methods, Jacobs University Bremen, Bremen, Germany; 2 FOM Hochschule für Oekonomie & Management, Bremen, Germany; 3 La Trobe University, Bendigo, Australia; University of Macau, MACAO

## Abstract

The constructive alignment (CA) of university teaching is designed to encourage students to adopt a deep learning approach, which supports meaningful learning. The evidence is mixed, however, with some studies showing that students may adopt a surface approach even when teaching promotes deep learning. To add to the understanding of the relationships between CA and learning approaches, we explored with quantitative measures two potential implications from prior qualitative research. First, we assessed with a novel questionnaire if students’ CA perceptions predicted adaptation towards a deep learning approach. Second, we explored relationships between deep approach adaptation and learning motivation, as well as perceived mental workload. 56 students from two second-year courses in different study programmes completed a learning approach questionnaire in the second (T_1_), seventh (T_2_), and the final fourteenth (T_3_) course week. At T_2_ and T_3,_ participants also rated the constructive alignment of the course, their learning motivation, and the mental workload. Regression analyses showed that ILO Clarity (i.e. being clear about the intended learning outcomes of the course) and receiving effective feedback were associated with a significant increase in deep approach scores from T_2_ to T_3_. That deep approach adaptation was in turn positively related to learning motivation in terms of higher ratings of one’s competence, the importance of high course performance, and course usefulness. Moreover, deep approach adaptation went with higher satisfaction of having accomplished one’s learning goals, but also with stronger feelings of insecurity and stress. Our findings suggest that students’ CA perceptions are meaningful predictors of learning approach adaptation that might eventually be developed into indicators of the effectiveness of CA implementation at the course level.

## Introduction

The mounting pressure on universities’ accountability for educational quality and effectiveness (see [1,2]) has increased interest in outcomes-based education that is intended to enhance higher education through identifying the gaps between intended and actual learning (cf. [3]). One of the major frameworks for outcomes-based education is the *Constructive alignment* (CA) approach (e.g. [4]), which may serve to develop holistic assessment systems [3] and to align curricula with intended graduate attributes (e.g. [5]). CA has therefore attracted substantial attention from a policy-making perspective and at the university and study programme levels [6].

Relatively little research, however, has evaluated CA from a student perspective and at the course level (cf. [7]) although such research is needed to maximise the benefits of CA. After all, the constructive alignment approach is premised on the assumption that students *actively construct* knowledge through their learning activities (e.g., [8]). The level of constructive alignment that students perceive might therefore differ from the level the instructor intended (cf. [9]). Consequently, research that includes students’ CA perceptions might therefore help gauge the effectiveness of CA implementation (cf. [10]). Against this backdrop, we investigated the relationships between *perceived* constructive alignment and learning behaviours in terms of students’ approaches to learning (hence: learning approaches). Building on extant qualitative research, we explored over the course of a semester how CA perceptions are related to students’ adapting their learning approaches from surface learning to deep learning and what the direct and indirect motivational consequences of such adaptation are in terms of learning motivation and mental workload.

### Constructive alignment and the adaptation of learning approaches

Constructive alignment is intended to enhance the quality of teaching and learning and hinges on defining the outcomes students are intended to achieve. These *intended learning outcomes* (ILO) nominate specific actions students are supposed to perform. They may be expected, for instance, to learn to “critically discuss the limitations of Self-Determination Theory” in an introductory course on motivation theories. In an advanced course on motivation, students may be supposed to “Generate from Self-Determination Theory solutions to a specific disciplinary problem in the workplace”. Different as those ILO may be in terms of complexity and difficulty, students will usually have to use several learning strategies in concert to process the content. They make, for instance, connections between the various pieces of information presented to them and with their background knowledge (i.e., generate inferences; [11,12]), examine the logic of arguments or usefulness of evidence [13], and monitor their understanding [14,15]. From the perspective of the Student Approaches to Learning framework [11,16], such learning behaviours reflect a student’s *deep approach to learning*, which can be conceptualised as an instance of self-regulated learning [8,17]. A deep learning approach is related to high-quality learning outcomes and study success [18,19]. Students adopting a *surface approach*, in contrast, focus on memorising and reproducing fragmented units of information [15,20,21], usually engage in only little reflection on their learning [22], and have difficulties with new ideas [23].

Constructive alignment is therefore meant to encourage a deep approach and to discourage a surface approach [24,25], building on the assumption that learning approaches reflect a personal preference rather than a trait. They may therefore change within students depending on the context [26,27]. Öhrsted and Lindfors [28], for instance, found that some students adopted course-specific approaches in parallel courses, i.e., in the same semester, such that they pursued a deep approach in one course, but a more surface approach in another course. To promote deep learning, teaching-learning activities (TLA) are aligned with the ILO so that what is taught is directly relevant for what students need to learn and so that students can literally practise the actions defined in the ILO. Finally, aligning assessment tasks (AT) with both ILO and TLA lets students demonstrate the achieved level of learning.

The evidence on the relationship between CA and learning approaches is mixed, however. Kember, Charlesworth, Davies, McKay, and Stott [29] report several case studies showing a positive association of CA and a deep learning approach. In one of those studies, for instance, the re-design of one study programme was evaluated. Students had in their first year been introduced to the different learning approaches and had identified in an initial assessment their own approach. Students’ deep approach scores were assessed at the beginning of the first, second, and third year, showing a linear increase from the first to the third year. As previous assessments of other study programmes had shown deep scores to *decrease* across study years, the increase in that case study was taken to reflect the fact that students had been given a variety of learning experiences that sought to promote ‘rich’, meaningful learning [29]. More recently, Rodriguez and Cano [30] found in a longitudinal study that between the first and final years of studies, students’ learning approaches moved towards depth. Wang, Su, Cheung, Wong, and Kwong [31] compared in a pre-post design several courses of two study programmes. They found that deep learning scores increased in students enrolled in the programme that was rated as more constructively aligned, relative to the programme deemed lower in CA. Reversely, surface learning approach scores went up for students in the ‘low-CA’ programme, but not the ‘high-CA’ programme.

In one of the studies that yielded negative evidence, Balasooriya, Toohey, and Hughes [32] had students report their learning approaches in a course that had been re-designed to enhance deep learning. Prior to that course, participants indicated the learning approach they had usually adopted in the previous courses of their programme. The authors obtained “unexpected patterns” such that, as supported by the course design, a subgroup of students adopted a deep approach. Another subgroup, however, rather used a surface approach although they had indicated having been deep approach learners in prior courses. Still another group displayed virtually no adaptation of their learning approach although the course in question differed markedly from those students’ prior courses. Similarly, Pang, Ho, and Man [33] showed that Outcomes-Based Teaching and Learning (OBTL) impacted on the learning approaches of undergraduate business students by tracking students’ scores after a semester’s exposure to outcome-based teaching. Yet, whilst the number of students adopting a surface approach nearly doubled, no significant change was found in the number of students endorsing a deep approach. Some students had even changed from the deep approach to the surface approach that they had initially favoured the least. Finally, counter to the expectation that students would adopt a deep approach through their course of study, Habel [34] found no changes in learning approaches in one course over the semester, even though that course had been designed in accordance with constructive alignment principles.

### Learning approaches as indicators of CA implementation quality

Taken together, there is some support for the idea that CA promotes a deep learning approach, but this positive effect does not seem to be guaranteed. Even when the learning environment promotes meaningful learning, students may choose to keep or even return to a surface approach. These mixed findings might reflect that CA was better implemented in some courses than in others. After all, students’ perceptions of their teaching–learning environment are related to their learning approaches [35,36] and students are more likely to adopt a deep approach the higher they rate teaching quality [36,37]. By that logic, a high proportion of surface-approach students would indicate quality problems and in principle, assessments of students’ learning approach adaptation could serve as indicators of CA effectiveness and teaching quality (cf. [10]).

Yet, two limitations of extant research render implications for CA quality management tentative. First, a-priori indicators of CA, albeit theory-based, were used in most studies, derived from analyses of programme descriptions, syllabi and/or from conversations with instructors. In case students’ CA perceptions were collected, those data were qualitative. Second, whilst students’ learning approaches proper were assessed with quantitative questionnaires, qualitative methods were used to explore the relationships between learning approach adaptation and motivation. Findings on students’ CA perceptions and the motivational consequences of learning approach adaptation are therefore difficult to compare between studies, which limits generalisation. On this background, we sought to contribute to research in a CA quality management direction by exploring with quantitative measures two potential implications from prior qualitative research. First, we assessed with a novel questionnaire students’ perception of constructive alignment. Second, we explored the motivational consequences of adaptation towards a deep learning approach in terms of learning motivation and of mental workload.

Our decision to assess students’ perceptions of constructive alignment builds on Balasooriya et al. [32] and Pang and colleagues’ [33] findings. In the latter study, students mentioned in focus groups as the most distinctive features of outcome-based teaching and learning (OBTL) the precision of the intended learning outcomes and the transparent assessment criteria. Although OBTL is concerned with aligning all aspects of teaching (ILO, contents, their delivery and learning activities, and assessments), students did not remark they saw the alignment of teaching-learning activities with ILO and/or assessments as important features of OBTL. Balasooriya et al. [32] concluded from their qualitative interviews that whether students adopted a deep or surface approach in response to the re-designed course had been co-determined by students’ perception of specific curricular features. As different students perceived the same course in different ways, course characteristics that were established in the literature as generally encouraging deep approaches promoted a deep approach in some students, while they stimulated surface approaches in others. Taken together, these findings suggest that rather than being perceived as a single, unitary course characteristic, the individual CA dimensions might have distinct, separately identifiable effects on students’ learning experience and eventually their learning approaches. Going by Pang et al.’s [33] findings, well-defined ILO and closely aligned AT might be particularly pronounced drivers of learning approach adaptation that help students gauge the usefulness of a deep learning approach. Therefore, we put forward our Research question 1: Do the dimensions of CA perceptions contribute differentially to students’ adaptation towards a deep learning approach (hence: deep approach adaptation)?

As a second potential implication from those studies, deep approach adaptation might have both direct and indirect effects on student motivation. Speaking to direct effects, students who moved towards deep learning reported that seeing the relevance of what they were doing made learning easier and felt the active nature of the course was a motivation for learning [32]. Similarly, the outcomes-based course design appeared to give students a clearer picture of their learning progress and next steps, thus positively guiding their development and encouraging some students to study topics beyond the scope of the lectures [33]. Indirect effects on motivation may arise from students’ perceptions of context characteristics such as the workload and task complexity associated with learning [38]. Illustrating negative effects, some of the students who kept or even returned to a surface approach perceived the reduction in lecturing and the increase in self-directed work that CA came with as less motivating and as “too much of an effort”, whereas getting information “given to you by some lecturer” would be much easier [32]. Also, some students remarked that given their time constraints, they could only “rely on reciting”, thus abandoning the more complex and costly learning approach [33]. Whilst neither study directly addressed positive effects, Kyndt, Dochy, Struyven and Cascallar’s [38] findings suggest that a deep approach may be associated with lower mental workload perceptions. Students in a regular university course worked individually on several assignments that had been constructed and pre-tested to induce specific levels of mental workload, which comprises one’s perceptions of the mental, temporal and motivational demands [39] of a task. Mental demands refer to task complexity and required mental activity (e.g., deciding, calculating, remembering, searching). Temporal demands concern perceived time pressure and motivational demands are perceptions how hard one had to work to accomplish a given level of performance. After each assignment, students completed a questionnaire on the learning approach they had adopted when they worked on that assignment and on perceived workload. Even for high-workload assignments, students with a deep approach reported lower mental demands of the respective assignments than students with a surface approach to the same assignment. More generally, a negative correlation emerged between a deep learning approach and perceived workload such that higher deep approach scores went with lower workload ratings. Conversely, higher surface approach ratings were associated with higher perceived mental demands [38]. On this background, we were interested in the relationships between deep approach adaptation and direct and indirect effects on student motivation. We assessed students’ learning approaches, learning motivation, and workload perceptions at three points in time over the course of one semester to answer Research question 2: Is deep approach adaptation associated with an increase in learning motivation? and Research question 3: Is deep approach adaptation associated with a decrease in perceived workload?

## Materials and methods

Our survey of students in one second-year undergraduate course from each of two bachelor’s programmes was designed to predict from students’ perceptions of four dimensions of constructive alignment the adaptation of learning approaches towards a deep approach. Also, we assessed if learning approach adaptation predicted changes in learning motivation and perceived mental workload.

### Sample and procedure

We invited all students in a course on *Supply Chain Management* and on *Mass Media in Digital Context* at Jacobs University Bremen. The study was conducted in compliance with the WMA Declaration of Helsinki, as well as the standards of Jacobs University’s Internal Review Board. Prior to completing the questionnaires, participants were informed in writing that the data from this study would be used in a project on teaching quality. Participants learnt that they would be asked to share their impressions of course characteristics (e.g., course goals, teaching methods), as well as their general attitudes towards learning and studying and that it would be studied how those characteristics contribute to students’ motivation. Furthermore, participants were informed that their participation was voluntary and that it would in no way affect their course grades if they chose not to participate. Participants were offered to enter a prize draw of four Amazon® gift vouchers of € 25 each by completing all three survey waves (see details below) and providing an email address in the final survey. To ensure participants’ anonymity, no personal information was collected, except for age and gender. Participants’ data were matched across waves by means of an alphanumeric code that we instructed participants to generate from family members’ names and birth dates.

Both courses comprised 14 weekly appointments of 150 min. each. Students completed questionnaires in the second (T_1_), seventh (T_2_), and the final fourteenth (T_3_) class. Questionnaires were handed out some 15 min. before the class ended and collected right after class. From the initial sample of 98 students (31.6% female, mean age 21.6 years, SD = 2.71), we retained all students who had completed all three questionnaires for a final sample of 56 students (21.4% female, mean age 19.1 years, SD = .84, response rate 57.1%). Students were from various majors (e.g., General Economics and Management, Industrial Engineering, International Business Administration, Logistics).

### Measures

Except for the mental workload questionnaire, all items were rated on Five-Point Likert scales. As English is the language of instruction at Jacobs University, we used all questionnaires in their original English versions.

At T_1_, we assessed students’ use of surface and deep approaches to learning with 20 items from the *Revised Two-Factor Study Process Questionnaire* (R-SPQ-2F; [26]). There were five items for each of the four subscales of *Deep Motive* (sample item: “I find that at times studying gives me a feeling of deep personal satisfaction.”), *Deep Strategy* (sample item: “I test myself on important topics until I understand them completely.”), *Surface Motive* (sample item: “My aim is to pass a course while doing as little work as possible.”), and *Surface Strategy* (sample item: “I learn some things by rote, going over and over them until I know them by heart even if I do not understand them.”). As demographic data, we collected participants’ major, age, and gender.

As an assessment of their motivation, students completed 16 items from the Intrinsic Motivation Inventory (IMI; [40,41]), with four items for each of the subscales of *Enjoyment* (sample item: “I think this class is very interesting.”), *Competence Self-Perception* (sample item: “I am pretty skilled at doing the tasks for this class.”), *Usefulness* (sample item: “I believe doing the activities in this class are beneficial to me.”), and *Importance* (sample item: “It is important to me to do well at the tasks in this class.”). Students filled in the R-SPQ-2F and the IMI at T_2_ and T_3_ as well.

At T_2_ and T_3_, students rated the constructive alignment of the course with the Constructive Alignment Questionnaire (CALEQ; [42]) that comprised five items for each of the four dimensions of *ILO Clarity* (sample item: “I had a clear idea of what I was supposed to learn.”), *Alignment of Teaching-Learning Activities* (TLA Alignment; sample item: “I was provided a variety of activities that helped me learn what I was supposed to learn.”), *Alignment of Assessment Tasks* (AT Alignment; sample item: “The assessment tasks addressed what I was supposed to learn.”), and *Feedback Effectiveness* (sample item: “I received feedback that was clear and specific to what I was supposed to learn.”).

Also, students indicated on the NASA Task Load Index (NASA-TLX; [39]) the mental workload they perceived from learning in that class. To each of the dimensions of *Mental Demand* (“Has this course been easy or demanding, simple or complex?”), *Temporal Demand* (“Has the learning been slow and leisurely or rapid and frantic?”), *Frustration Level* (“How insecure, stressed or annoyed versus secure, relaxed or content have you felt during the learning?”), *Performance Satisfaction* (“How satisfied have you been with your performance in accomplishing the goals of the learning activities?”), and *Required Effort* (“How hard did you have to work to accomplish your level of performance?”), students assigned a value between 0 and 100 percent.

## Results

We analysed with hierarchical regression analyses the data from 56 participants using the IBM SPSS® Statistics software (Version 26.0). In an initial data screening, we found no violations of regression assumptions or multicollinearity (all tolerance scores > .25). Cronbach’s α as an indicator of scale reliability was greater than .70 for all scales, warranting the quantitative analyses reported below. A multivariate analysis of variance with *study programme* as factor and the mean scores on the dimensions of *perceived constructive alignment*, *learning demands*, and *learning approach* did not reveal significant differences between participants as a function of their programme (F< 1, ns.). We therefore collapsed the data from the two courses for all analyses. Also, we included age, major, and gender as controls but do not report these data below, as none of the controls yielded any significant effects. The level of statistical significance was set at α< .05 for all analyses.

### Learning approach adaptation

As pre-requisites to the main analyses, we assessed changes in students’ learning approaches over the semester. In repeated-measures analyses of variance, we compared the mean deep and surface approach scores, respectively, between T_1,_ T_2_, and T_3._ For the approach scores, we summed up a participant’s ratings on deep-strategy items and deep-motive items, as well as surface-strategy and surface-motive item for the deep and surface approach scores, respectively. Table 1 summarises the results.

**Table 1:** Prediction of deep approach adaptation from constructive alignment perceptions.

		Mean	SD	F_(2,54)_
Deep approach score	T_1_	30.00	4.96	14.67^a^
	T_2_	29.91	5.99	
	T_3_	35.90	6.78	
Surface approach score	T_1_	25.67	5.68	1.69^b^
	T_2_	24.59	6.13	
	T_3_	23.61	5.58	

The range of each approach score was 10.00–50.00.

^a^Statistically significant F-value.

^b^Statistically non-significant F-value.

The analysis revealed a significant increase in students’ deep approach scores, whereas the mean surface approach score decreased from T_1,_ to T_3_, albeit non-significantly so. In both scores, there was no significant change from T_1_ to T_2_, indicating that students adapted their learning approaches only in the second half of the semester, i.e. from T_2_ to T_3._

### Constructive alignment perceptions and deep approach adaptation

Addressing our first research question of differential contributions of constructive alignment perceptions to deep approach adaptation, we regressed the four dimensions of CA perceptions (ILO Clarity, TLA Alignment, AT Alignment, and Feedback Effectiveness) at T_3_ onto deep approach adaptation, computed as the difference in deep approach scores between T_2_ and T_3._ Table 2 displays the regression model summary and standardised ß-coefficients with their T-values for the CA perceptions.

**Table 2:** Relationships between perceived constructive alignment and deep approach adaptation.

CA dimension			Criterion	Model summary		
	ß	T		R	R^2^	SE
ILO Clarity	.49	2.94^a^	Deep approach adaptation	.494	.244	8.333
TLA Alignment	-.12	-.57^b^				
AT Alignment	-.17	-.81^b^				
Feedback	.31	2.13^a^				

^a^Statistically significant T-value.

^b^Statistically non-significant T-value.

We found no overall effect of CA perceptions as not all CA dimensions were associated with deep approach adaptation. *ILO Clarity* (i.e. being aware of and clear about a course’s intended learning outcomes) was positively associated with an increase of deep approach scores, just as receiving effective feedback (*Feedback Effectiveness*) went with increasing deep approach scores. Neither the degree to which teaching-learning activities were seen as aligned with ILO, i.e. *TLA Alignment*, nor students’ perceptions of the assessment tasks being aligned with ILO and TLA (*AT Alignment*) predicted changes in deep approach scores.

### Deep approach adaptation, motivation, and perceived workload

Assessing direct and indirect effects on motivation, we determined if deep approach adaptation is associated with an increase in student motivation (Research question 2) and with a decrease in perceived workload (Research Question 3). We regressed the T_2_-T_3_ deep approach adaptation score onto the T_2_-T_3_ difference scores of the four IMI dimensions of competence self-perception, enjoyment, importance, and course usefulness. Furthermore, we regressed the deep approach adaptation score onto the T_2_-T_3_ difference scores of the five indicators of mental demand, temporal demand, frustration, satisfaction, and required effort. Table 3 lists the regression model summaries and standardised ß-coefficients with T-values for all motivation and workload dimension.

**Table 3:** Relationships between deep approach adaptation, student motivation, and workload.

Criteria			Predictor	Model summaries		
	ß	T		R	R^2^	SE
Competence	.41	3.34^a^	Deep approach adaptation	.414	.172	2.958
Enjoyment	.04	.33^b^		.045	.002	2.475
Importance	.31	2.39^a^		.310	.096	3.221
Course usefulness	.40	3.18^a^		.310	.096	3.333
Mental demand	-.09	-.72^b^	Deep approach adaptation	.099	.010	105.3
Temporal demand	.19	1.38^b^		.188	.035	33.51
Frustration	.31	2.34^a^		.309	.096	37.47
Performance satisfac.	.49	4.10^a^		.494	.244	20.69
Required effort	.03	.22^b^		.030	.001	34.05

^a^Statistically significant T-value.

^b^Statistically non-significant T-value.

As a direct effect, deep approach adaptation was associated with higher perceptions of one’s competence, of the importance of performing well, and of the course being useful. The relationship with course enjoyment was not significant. Indicating indirect effects, deep approach adaptation predicted performance satisfaction such that adaptation went with higher satisfaction ratings of having accomplished one’s learning goals. Also, adaptation predicted students’ frustration levels. Importantly, the correlation was positive, suggesting that adaptation may come with higher levels of feeling insecure and stressed.

## Discussion

Constructively aligned teaching is designed to encourage students to adopt a deep learning approach, which supports meaningful learning. Several studies yielded evidence of that positive relationship between CA and a deep learning approach. Some studies showed, however, that students may adopt and keep a surface approach even when teaching promotes deep learning. These mixed findings might reflect that CA was better implemented in some courses than in others. Research on the relationships between CA and students’ learning approaches may therefore lead to indicators of CA effectiveness and teaching quality. Our exploratory study was meant to help establish an approach for such research by exploring with quantitative measures two potential implications from prior qualitative research.

First, we collected with a novel questionnaire students’ perception of four dimensions of constructive alignment. Our findings suggest that those CA perceptions contribute selectively to deep approach adaptation. Higher ratings of ILO clarity and feedback effectiveness went with more learning approach adaptation, whilst the alignment of teaching-learning activities and of assessment tasks was unrelated to adaptation. As a second implication, we addressed direct and indirect relationships between deep approach adaptation and motivation. Indicating a direct relationship, adaptation was related to increases in competence self-perceptions, the value of effort, perceived course usefulness, and satisfaction with one’s performance, but not to increased enjoyment of the course. A positive correlation emerged between adaptation and increased feelings of insecurity and stress, whilst adaptation was unrelated to changes in the perceived mental and temporal demands.

In sum, students’ CA perceptions appear to be significant predictors of learning approach adaptation. Our findings are consistent with those of Balasooriya et al. [32] and Pang et al. [33], which suggest that rather than either being fully aligned or not at all, courses may differ in the degree to which they are perceived as constructively aligned. Complementing extant evidence of motivation being an antecedent of learning approaches (e.g., [38,43]), our data suggest that motivation may also be a consequence of the latter such that adopting a deep approach may come with the ‘benefit’ of heightened competence self-perceptions but also with ‘costs’ in the form of experiencing increased workload. To the extent that instructors can positively influence both costs and benefits through the way they implement CA principles, research on the relationships between CA perceptions, learning approach adaptation and its motivational consequences might help develop novel ways of managing CA implementation quality.

This study was intended to be exploratory and yield suggestions for further research, rather than for instructional practice. Still, the small sample size and the fact that we collected data in only two courses are limitations that need to be addressed in future research with larger samples. Given a mean observed effect size of .79 (Cohen’s d for tests of means differences) in earlier research [29–31]–no statistical tests were reported in [32,33]–that indicates strong effects [44], it seems safe to assume a medium-level population effect size for CA-learning approach relationships. At an assumed Cohen’s f^2^ of .15, representing a medium-level correlational effect, the statistical power of our tests ranged from .81 (single-predictor models of motivational outcomes) and a mere .58 (four-predictor model of deep approach adaptation), i.e., were at or even far below the level of .80 that is usually considered acceptable [44]. Future studies with a similar design will require at least 129 participants to achieve a power level of .95 [45]. Beyond statistical power, larger samples allow for comparisons between, for instance, subgroups of international students. Given its steady increase in the past 20 years [46], international student mobility has gained importance as a driver of international scientific co-operation networks [47]. Universities will therefore seek to counteract the decline in student mobility caused by the COVID-19 pandemic by, for instance, optimising their classroom teaching to meet the needs of international students and incentivise them to cross borders for the purpose of study [47]. Investigating the role of educational culture would be a worthwhile topic in this regard. For instance, learning beliefs and classroom behaviours have been shown to differ markedly between North American and European on the one hand and East Asian students on the other hand [48], leading to different perceptions of the ease of engaging in certain classroom communication styles, which is in turn associated with higher levels of satisfaction with one’s learning and with better grades [49]. In a similar fashion, engagement in constructively aligned learning activities and the workload they induce might partly depend on the learning culture students come from. Identifying such differences in future research could help instructors adjust their teaching-learning activities and/or assessment tasks.

Further research is also needed on the context sensitivity of CA perceptions. Two questions in this regard are (a) why students adapted their learning approaches only in the second semester half and (b) why the alignment of teaching-learning activities (TLA) and assessment tasks (AT), respectively, did not predict that adaptation. As a tentative answer, we suggest this pattern reflects the context-sensitive, interactive nature of the four dimensions of CA perceptions. The assessments (mid-term exam and final exam) were administered in the second semester half. The late adaptation might thus reflect a backwash effect (see [50]) such that the exams influenced what and how students learned by letting them realise that deep learning would be useful. In that view, the ‘assessment backwash’ would have been a second source of information. It may have confirmed students’ initial ILO-based perception (ILO had been announced at course start) that deep learning would be required, suggesting an additive interaction between ILO clarity and feedback effectiveness. Perceptions of TLA and AT alignment, on the other hand, might be necessary but not sufficient conditions of deep approach adaptation. If those alignment perceptions are above a certain level, ILO clarity supports adaptation towards deep learning but may not do so in case of misalignment. Although merely speculative, that perspective on CA perceptions seems plausible considering extant findings. Our results fit with Pang and colleagues’ [33] finding that students perceived clear ILO and transparent assessment criteria, but not TLA or AT alignment as the most distinctive features of outcome-based teaching and learning. Also, in addition to the perceptions of a specific course, the general learning experience that they accumulate across different courses can influence students’ approaches to learning (see Baeten et al. [51] for a review). This would help explain the late adaptation we found. Students may have experienced in some of their courses that learning outcomes were not aligned with assessments. Therefore, they ‘waited’ to gauge from the mid-term exam and the feedback they received on and from it which learning approach might be most suitable. This seems plausible given the adverse motivational consequences of the ‘wrong’ learning approach that Leber and colleagues [52] found. They compared two parallel versions of a course (same lecturer, topics, learning materials, students from same year of study and programme). Students were informed at course start of the ILO, which required deep learning (e.g., being able to explain the theoretical concepts behind several psychological models, applying those models to optimise a specific learning environment). Also, an assessment on those contents was announced for a later session. In one group, that assessment was announced as an essay exam, which required deep understanding of the learning contents. In the other group, a fact-oriented multiple-choice test was announced, which primarily required recalling information, i.e., surface learning. Students in that misaligned group (‘deep’ ILO, surface assessment) reported significantly higher levels of feelings of pressure and tension than the aligned group (deep ILO, deep assessment). At the same time, the aligned group indicated higher feelings of competence.

Context sensitivity also appears to be an issue for future research concerning the motivational consequences of learning approaches. We cannot offer a compelling explanation why deep approach adaptation was unrelated to several direct and indirect motivation indicators. Further research is therefore needed into potential context-sensitive patterns that may underlie our results. Deep approach adaptation did not go, for instance, with higher levels of enjoyment. At the same time, fitting with the findings of Leber and colleagues [52], adaptation was positively related to competence self-perception and perceived course usefulness. This seems plausible given the correlation between deep approach adaptation and students’ frustration levels. Adopting a deep approach may not ‘come easy’ and put students under some stress, which is likely to limit the joy the learning brings. Still, as it is intended to foster meaningful learning, a deep approach can contribute to feeling competent and perceiving the learning as useful. There is some evidence suggesting that the adoption of a deep learning approach partly depends on academic discipline and course topic (see [51]). Future research could therefore explore if the relationships between deep approach adoption, frustration levels, and course enjoyment play out differently in different learning environments.
